# Differences in the Biomarker Profile of De Novo Acute Heart Failure versus Decompensation of Chronic Heart Failure

**DOI:** 10.3390/biom11111701

**Published:** 2021-11-16

**Authors:** Sylwia Nawrocka-Millward, Jan Biegus, Magdalena Hurkacz, Mateusz Guzik, Marta Rosiek-Biegus, Ewa Anita Jankowska, Piotr Ponikowski, Robert Zymliński

**Affiliations:** 1 Institute of Heart Diseases, University Hospital, 50-556 Wroclaw, Poland; mateuszguzik23@gmail.com (M.G.); evitajankowska@gmail.com (E.A.J.); ppponikowski@gmail.com (P.P.); robertzymlinski@gmail.com (R.Z.); 2 Institute of Heart Diseases, Medical University, 50-556 Wroclaw, Poland; 3 Department of Clinical Pharmacology, Medical University, 50-556 Wroclaw, Poland; magdalena.hurkacz@umw.edu.pl; 4 Department of Internal Medicine, Pneumology and Allergology, Medical University, 50-369 Wroclaw, Poland; martarosiek@gmail.com

**Keywords:** de novo AHF, acute decompensated chronic HF, heart failure, biomarkers

## Abstract

The perception of acute heart failure (AHF) as a single entity is increasingly outdated, as distinct patient profiles can be discerned. Key heart failure (HF) studies have previously highlighted the difference in both the course and prognosis of de novo AHF and acute decompensated chronic HF (ADHF). Accordingly, distinct AHF profiles with differing underlying pathophysiologies of disease progression can be shown. We compared a range of selected biomarkers in order to better describe the profile of de novo AHF and ADHF, including the inter alia—serum lactate, bilirubin, matrix metallopeptidase 9 (MMP-9), follistatin, intercellular adhesion molecule 1 (ICAM-1), lipocalin and galectin-3. The study comprised 248 AHF patients (de novo = 104), who were followed up for one year. The biomarker data of the de novo AHF and ADHF profiles was then compared in order to link biomarkers to their prognosis. Our study demonstrated that, although there are similarities between each patient profile, key biomarker differences do exist—predominantly in terms of NTproBNP, serum lactate, bilirubin, ICAM-1, follistatin, ferritin and sTfR (soluble transferrin receptor). ADHF tended to have compromised organ function and higher risks of both one-year mortality and composite endpoint (one-year mortality or rehospitalization for heart failure) hazard ratios (HR) (95% CI): 3.4 (1.8–6.3) and 2.8 (1.6–4.6), respectively, both *p* < 0.0001. Among the biomarkers of interest: sTfR HR (95% CI): 1.4 (1.04–1.8), NGAL_(log)_ (neutrophil gelatinase-associated lipocalin) HR (95% CI): 2.0 (1.3–3.1) and GDF-15_(log)_ (growth/differentiation factor-15) HR (95% CI): 4.0 (1.2–13.0) significantly impacted the one-year survival, all *p* < 0.05.

## 1. Introduction

Acute heart failure (AHF) is a multifaceted clinical condition with the potential to be life-threatening and requiring urgent treatment [1]. Although AHF is perceived as a single entity, it includes heterogenic populations of patients; thus, distinct patient profiles can be recognized [2]. The differences can be shown in several aspects of clinical presentation, such as: renal function, natriuretic response, volume status or neurohormonal activity [3,4,5,6,7,8]. We speculate that the pathophysiology underlying patients at various stages of the natural course of heart disease is different, at least in terms of the severity of some phenomena. Furthermore, the mechanisms that drive both the decompensation itself and poor outcomes may vary. This observation has critical practical implications, as the analyses of several key AHF clinical trials have shown differences in both the clinical course and prognosis of de novo and acute decompensated chronic heart failure (ADHF) [9,10,11].

We aim to provide a comprehensive picture of several selected biomarkers and compare them between patients experiencing their first episode of AHF (de novo AHF) with those who had heart failure diagnosed prior to hospital admission (ADHF).

## 2. Materials and Methods

### 2.1. Study Population

This was a single-center, observational study that was undertaken in the Centre of Heart Diseases, 4th Military Hospital, Wroclaw, Poland between January 2016 and September 2017. AHF was defined according to the European Society of Cardiology (ESC) guidelines criteria, and events were classified as new onset without a prior history of heart failure (de novo AHF) and ADHF [12,13]. All adult patients (≥18 years) hospitalized with AHF as the primary cause of hospitalization treated with intravenous furosemide at admission and those willing to participate (by signing an informed consent form) were enrolled. Exclusion criteria included the following: cardiogenic shock, diagnosis of acute coronary syndrome, known severe liver disease, end-stage renal disease requiring renal replacement therapy and evidence of infection. Patients were treated in accordance with the attending physicians’ recommendations of the ESC guidelines.

### 2.2. Study Design

After admission to hospital, detailed information concerning patient demographics (including history of heart failure), clinical history, comorbidities, previous therapies and physical findings, were collected. Dyspnea was measured with the use of a self-reported 10-point Likert scale at the time of hospital admission, where 0 meant ‘lack of dyspnea’ and 10 points meant ‘dyspnea of the worst severity/maximal dyspnea’. Clinical assessments alongside venous blood were obtained at baseline and the following subsequent time points: day 1 and day 2 and discharge. The samples were also collected, centrifuged and frozen (at −70 °C) for additional prespecified analyses. A current literature review was undertaken, and biomarkers were selected on the basis of their relation to the pertinent heart failure (HF) pathophysiological pathways. The selected biomarkers were involved in inflammation (CRP, IL-6, IL-22 and WBC); liver function (AST, ALT and bilirubin); perfusion and congestion (NTproBNP and lactate); iron status (Fe, total iron binding capacity, sTfR and ferritin) and cardiac remodeling (MMP-3, follistatin, selectin, lipocalin, PF4, myostatin, ICAM-1, GDF-15 and galectin-3). The properties of each marker and evidence in HF have been described elsewhere [14,15,16,17,18,19,20,21]; a brief description of each biomarker can be found in the Supplementary Materials. Information regarding the biomarkers of interest was not available to the treating physicians, and neither clinical nor therapeutic decisions were based on these results.

### 2.3. Laboratory Measurements in Peripheral Blood

Laboratory parameters were assessed using the standard methods in our laboratory, including plasma NTproBNP (N-Terminal Pro-B-Type Natriuretic Peptide) (method: immunoenzymatic, Siemens, Marburg, Germany) and troponin (TNI) (method: immunoenzymatic, one-dimensional RxLMax, Siemens). The serum sTfR (mg/L) was measured from plasma frozen at −70 °C using immunonephelometry (Siemens Healthcare Diagnostics, Inc., Deerfield, IL, USA).

The Quantikine ELISA Immunoassay kit (R&D Systems, Inc., Minneapolis, MN, USA) was used to determine the levels of the remaining markers of interest. This assay employs the quantitative sandwich enzyme immunoassay technique. These were the following proteins: GDF-15 (also known as macrophage inhibitory cytokine-1 (MIC-1) (*n* = 79), CXL4/PF4 (Platelet Factor 4), follistatin (FS), MMP-9 (matrix metalloproteinases—gelatinase) (*n* = 159), lipocalin-2 (NGAL) (*n* = 159), myostatin (GDF-8), E-Selectin (CD62E), ICAM-1 (CD54 allele-specific), Il-6 and Il-22 (*n* = 159). The Synergy/HTX multi-mode reader analyzer was used for the measurement of absorbance. This research was carried out in the laboratory of the Department of Clinical Pharmacology at Wroclaw Medical University.

### 2.4. Study Outcomes

The clinical endpoints of the study were:

1. In-hospital mortality;
2. One-year mortality;
3. Composite endpoint of one-year mortality and rehospitalization for heart failure.

### 2.5. Clinical Follow-Up

Discharged patients were tracked at the heart failure clinic for at least one year. Information regarding rehospitalizations and survival status was obtained either directly from patients or their relatives (telephone contact), from the HF clinic database, from the hospital system or from the national citizen registry by the investigators, who were blinded to the biomarker results. No patient was lost to follow-up.

### 2.6. Statistical Analysis

Continuous variables with a normal distribution were described using means ± standard deviation, variables with skewed distribution were described by medians with (upper and lower quartiles) and categorized variables were given as numbers and percentages. The statistical significance of the differences between groups was assessed using the *t*-test, Mann–Whitney *U* test or Kruskal–Wallis test. The normality of the distributions for these variables was evaluated by three different statistical tests: the Kolmogorov–Smirnov test, Lilliefors test and the W Shapiro–Wilk test. The Cox proportional hazards models were used to calculate the hazard ratio (HR) with the corresponding 95% confidence interval (95% CI) for all-cause mortality. Multivariable analyses were adjusted for: age, ejection fraction, systolic blood pressure at admission, hemoglobin, NTproBNP and blood urea nitrogen. Kaplan–Meier survival curves were constructed to demonstrate the survival. *p* < 0.05 was considered statistically significant. Statistical analyses were performed using STATISTICA 12 (StatSoft Polska Sp. z o.o., Krakow, Poland).

## 3. Results

### 3.1. Baseline Characteristics

The study population consisted of 248 patients, 182 (73.4%) male and with a mean (±SD) age of 70.1 ± 12.6 years. The mean (±SD) Left Ventricle Ejection Fraction (LVEF) was 37 ± 14%. De novo AHF was observed in 104 (41.9%) patients and ischemic HF etiology in 124 (50%) patients. The mean systolic blood pressure, serum Na^+^, hemoglobin and serum creatinine on admission were 134 ± 31 mmHg, 139 ± 4 mmol/L, 13.3 ± 2.0 g/dL and 1.36 ± 0.52 mg/dL, respectively. The median (upper and lower quartiles) plasma concentrations of NTproBNP and troponin I were 5618 (3431–11,750) pg/mL and 0.06 (0.03–0.16) ng/mL, respectively. The most common comorbidities and HF risk factors are presented in Table 1. Hypertension was the most frequent comorbidity in both de novo AHF and ADHF at 79.8% and 82.7%, respectively (Table 1). For ADHF patients, the median time since the first diagnosis of HF was 4 (1–7) years.

### 3.2. Comparison of Clinical and Basic Laboratory Characteristics

Patients with ADHF presented significantly lower LVEF 35 ± 1 compared to 40 ± 13% amongst the de novo AHF patients, *p* < 0.05. Higher levels of systolic and diastolic blood pressure on admission were observed in the de novo AHF patients (145 ± 33 vs. 126 ± 27 mmHg and 85 ± 16 vs. 75 ± 15 mmHg, all *p* < 0.001,respectively), alongside a higher average heart rate (95 ± 25 vs. 87 ± 23 bpm, *p* = 0.007) in comparison to ADHF. There was no statistically relevant difference in the length of hospitalization and reported dyspnea between both groups. Lower levels of sodium were observed amongst ADHF patients (138 ± 5 vs. 140 ± 4 mmol/L, *p* < 0.01) (Table 1).

### 3.3. Comparison of Comorbidity and Risk Factors

Of the comorbidity and risk factors we observed in the study population, CAD was significantly more prevalent in ADHF patients (69.4%) than in the de novo group (38.5%), *p* < 0.01. Conversely, hypertension was more frequent in the de novo group (82.7% vs. 77.8%), albeit not significantly, *p* = 0.34 (Table 1).

**Table 1:** Baseline characteristics of the population.

Parameter	Population	De Novo AHF (*n* = 104)	ADHF (*n* = 144)	*p*
Sex (male)	182 (73.4%)	68 (65.4%)	114 (79.2%)	0.020
Age (years)	70.1 ± 12.6	71.0 ± 12.6	69.4 ± 12.6	0.310
Heart rate (beat/minute)	90 ± 24	95 ± 25	87 ± 23	0.007
Systolic blood pressure at admission (mmHg)	134 ± 31	145 ± 33	126 ± 27	<0.001
Diastolic blood pressure at admission (mmHg)	79 ± 16	85 ± 16	75 ± 15	<0.001
Left Ventricle Ejection Fraction (%)	37 ± 14	40 ± 13	35 ± 14	0.039
Ejection Fraction (≤40%)	159 (64%)	54 (52%)	105 (73%)	<0.001
Dyspnea at admission (points)	8.1 ± 2.2	8.2 ± 2.4	7.9 ± 2.1	0.257
Ischemic etiology of heart failure	124 (50%)	35 (33.6%)	89 (61.8%)	<0.001
Blood Count:				
Hemoglobin (g/dL)	13.3 ± 2.0	13.3 ± 1.9	13.3 ± 2.0	0.966
WBC (G/L)	9.2 ± 4.5	9.5 ± 3.9	9.1 ± 4.8	0.526
PLT (G/L)	210 ± 88	209 ± 86	210 ± 90	0.948
AST (IU/L)	28 (22–41)	31 (24–49)	26 (21–39)	0.034
ALT (IU/L)	31 (21–56)	34 (23–60)	30 (21–48)	0.218
Bilirubin (mg/dL)	1.4 ± 1.3	1.2 ± 0.8	1.6 ± 1.6	0.011
Na (mmol/L)	139 ± 4	140 ± 4	138 ± 5	0.013
Creatinine (mg/dL)	1.36 ± 0.52	1.30 ± 0.55	1.41 ± 0.49	0.095
Blood Urea (mg/dL)	59.7 ± 31.3	56 ± 34	62 ± 29	0.132
C-reactive protein (mg/L)	7.7 (4.1–18.9)	7.2 (3.4–18.6)	7.75 (4.4–19.5)	0.541
NTproBNP(pg/mL)	5618 (3431–11,750)	5108 (2593–11,579)	5797 (3829–11,920)	0.06
Troponin I (ng/mL)	0.06 (0.03–0.16)	0.05 (0.02–0.18)	0.06 (0.03–0.14)	0.49
Systolic blood pressure at 24 h (mmHg)	122 ± 23	145 ± 33	126 ± 27	<0.0001
Creatinine at 24 h (mg/dL)	1.31 ± 0.49	1.3 ± 0.55	1.4 ± 0.48	0.10
Lactate on admission (mmol/L)	2.0 (1.5–2.6)	2.2 ± 0.9	2.3 ± 1.3	0.667
Lactate at 24 (mmol/L)	1.8 (1.5–2.4)	2.0 ± 0.6	2.3 ± 1.7	0.021
Length of hospitalization (days)	7 (5–9)	7 (5–8)	7 (5–11)	0.140
Comorbidity/risk factors:				
Coronary artery disease	140 (56%)	40 (38.5%)	100 (69.4%)	<0.001
Hypertension	198 (79.8%)	86 (82.7%)	112 (77.8%)	0.34
Cigarette smoking	111 (44.8%)	49 (47%)	62 (43%)	0.53
Diabetes mellitus	94 (37.8)	37 (36%)	57 (39.6%)	0.52
Chronic obstructive pulmonary disease	30 (12.1%)	12 (12%)	18 (13%)	0.82
Liver disease	22 (8.9%)	10 (9.6%)	12 (8.3%)	0.42

### 3.4. Administered Drug Class and Invasive Procedures—Before and during Hospitalization

Angiotensin-converting enzyme inhibitors (ACEI)/angiotensin receptor blockers (ARB), beta-blockers and mineralocorticoid receptor antagonists (MRA) were all significantly more frequently administered to ADHF patients (82% vs. 46%, *p* < 0.001; 74% vs. 41%, *p* < 0.005 and 35% vs. 9.6%, *p* < 0.001, respectively). During hospitalization, all patients received intravenous furosemide. Intravenous nitrates were administered more frequently to AHF de novo patients (61%) than ADHF patients (46%), *p* = 0.02. The opposite was observed for inotrope administration: 14% of ADHF patients were administered with inotropes, with only 6% of the de novo group receiving them, *p* = 0.03. The remaining drug classes and all invasive procedures were comparably utilized between both groups (Table 2).

### 3.5. Comparison of Selected Biomarkers—De Novo AHF and ADHF

No significant difference between selected biomarkers concerning inflammation (including CRP, IL-6 and IL-12) could be observed between the de novo AHF and ADHF groups (Table 3). Of the liver function tests we analyzed, the total bilirubin at admission was significantly higher in the ADHF group (1.6 ± 1.6 mg/dL) in comparison to the de novo AHF group (1.2 ± 0.8 mg/dL, *p* = 0.011). The majority of biomarkers associated with remodeling did not demonstrate significant differences between the two patient profiles. One exception here was ICAM-1, which was significantly lower in the de novo group 367.5 ± 152.4 vs. 416.7 ± 195.8 ng/mL, *p* = 0.035. Perfusion and congestion markers in patients with de novo AHF and ADHF differed—mean (±SD) lactate concentrations at discharge were 1.9 ± 0.6 and 2.1 ± 1.1 mmol/L. The median NTproBNPs at admission were 5108 (2593–11,579) and 5797 (3829–11,920) pg/mL, *p* = 0.06, and the median NTproBNPs at discharge were 2743 (1531.5–4798.5) and 3601 (2084–7284) pg/mL, *p* < 0.01, respectively.

In regard to the iron status, sTfR at both admission and discharge was lower in de novo AHF patients when compared to the ADHF group (admission—de novo AHF 1.8 ± 0.8, ADHF 2.2 ± 0.9 mg/L, *p* ≤ 0.001; discharge—de novo AHF 1.8 ± 0.8, ADHF 2.3 ± 1.1 mg/L, *p* = 0.001). Conversely, the ferritin levels were higher in the de novo AHF group: 203.1 ± 163.6 de novo vs. 156.6 ± 134.5 µg/dL ADHF, *p* = 0.025.

**Table 3:** Comparison of selected biomarkers of de novo AHF vs. ADHF.

Variable	De Novo AHF	ADHF	*p*
Infection/inflammation:			
C-reactive protein (mg/L)	7.2 (3.4–18.6)	7.8 (4.4–19.5)	0.541
IL-6 (pg/mL)	8.0 (1.1–21.7)	9.1 (0.5–20.0)	0.695
IL-22 (pg/mL)	9.0 (1.0–23.0)	5.0 (1.0–18.0)	0.328
WBC (G/L)	9.5 ± 3.9	9.1 ± 4.8	0.526
Liver function tests:			
AST at admission (IU/L)	31 (24–49)	26 (21–39)	0.034
ALT at admission (IU/L)	34 (23–60)	30 (21–48)	0.218
Total bilirubin at admission (mg/dL)	1.2 ± 0.8	1.6 ± 1.6	0.011
Perfusion and congestion markers:			
Lactate at admission (mmol/L)	2.2 ± 0.9	2.3 ± 1.3	0.667
Lactate at day-1 (mmol/L)	2.0 ± 0.8	2.2 ± 1.4	0.087
Lactate at discharge(mmol/L)	1.9 ± 0.6	2.1 ± 1.1	0.031
NTproBNP at admission (pg/mL)	5108 (2593–11,579)	5797 (3829–11,920)	0.06
NTproBNP at discharge (pg/mL)	2743 (1531.5–4798.5)	3601 (2084–7284)	0.01
Remodeling and other markers:			
MMP-9 (ng/mL)	383.1 ± 363.0	313.0 ± 265.6	0.162
Follistatin (pg/mL)	2739.5 ± 1952.6	2319.9 ± 1493.6	0.057
Selectin (ng/mL)	32.1 ± 15.7	34.1 ± 18.1	0.365
Lipocalin/NGAL (ng/mL)	82.3 ± 53.7	87.7 ± 54.4	0.542
PF4 (ng/mL)	6549.0 ± 3658.0	6833.9 ± 3132.2	0.513
Myostatin (pg/mL)	1818.8 ± 1029.5	1784.9 ± 1175.4	0.815
ICAM-1 (ng/mL)	367.5 ± 152.4	416.7 ± 195.8	0.035
GDF-15 (pg/mL)	4474.8 ± 1830.9	4752.1 ± 1453.7	0.478
Galectin-3 (ng/mL)	19.2 (13.7–33.8)	21.0 (12.2–32.1)	0.740
Iron status:			
Fe (µg/dL)	56.3 ± 33.7	55.6 ± 26.4	0.870
Total iron binding capacity (µg/dL)	336.6 ± 70.0	353.8 ± 67.6	0.068
sTfR at admission (mg/L)	1.8 ± 0.8	2.2 ± 0.9	<0.001
Ferritin (µg/L)	203.1 ± 163.6	156.6 ± 134.5	0.025

### 3.6. In-Hospital Mortality and Biomarkers of Interest

The in-hospital mortality was 4% (*n* = 10 patients). A comparison of the selected biomarkers revealed that, apart from the organ function markers (such as: Na^+^, creatinine, blood urea and NTproBNP), lactate 3.8 ± 2.8 vs. 2.1 ± 0.9 mmol/L and galectin 337.1 (18.3–109.7) vs. 19.8 (12.2–31.5) ng/mL were significantly higher among patients who died during hospitalization, all *p* < 0.05.

### 3.7. Comparison of Selected Biomarkers between Patients Who Experienced an Event (Death or Heart Failure Rehospitalization, Whichever Occurred First) and the One-Year Event-Free Group

Lower lactate levels at day 1 were observed in the event-free group 2.0 ± 0.6 vs. 2.3 ± 1.7 mmol/L, *p* = 0.021; likewise, the NTproBNP levels for the event-free group were lower at admission: 5080 (2944–9101) vs. 6312 (4083–13,944) pg/mL, *p* < 0.004 and at discharge: 2636 (1499–4802) vs. 4318 (2616–8210) pg/mL, *p* < 0.0005. NGAL differed significantly between profiles where we observed lower levels in the event-free group: 77.9 ± 51.8 in comparison to 95.7 ± 55.6 ng/mL, *p* = 0.039 in death or rehospitalized patients. Concerning the iron status, sTfR was lower in the event-free group compared to the death or rehospitalization group: 1.9 ± 0.7 and 2.2 ± 0.9 mg/L, *p* = 0.006, respectively (Table 4).

### 3.8. Differences in One-Year Outcomes between De Novo AHF and ADHF

**Figure 1:**
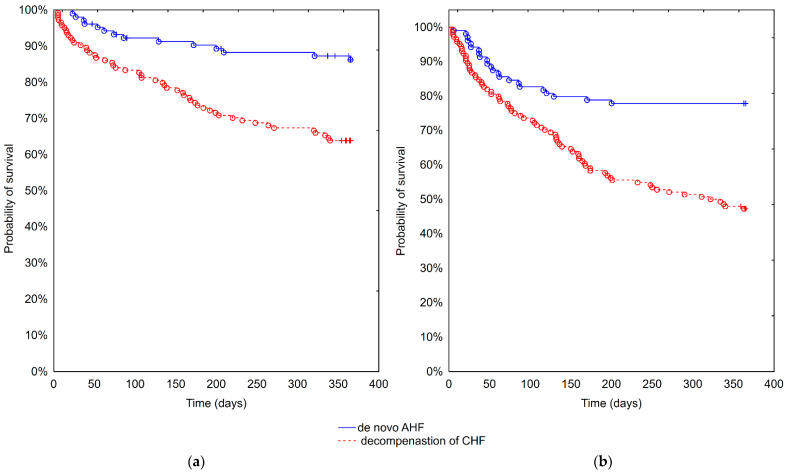
Kaplan–Meier curves comparing de novo AHF and ADHF. (**a**) Death. Log-rank *p* < 0.001. (**b**) Death or heart failure rehospitalization. Log-rank *p* < 0.001.

There were 66 (31.3%) deaths and 88 (41.7%) patients who experienced the composite study endpoint (death or rehospitalization for HF, whichever occurred first) within one year of observation. In the multivariable analyses, the ADHF group had a significantly higher risk of both endpoints, hazard ratios (HRs) (95% CI): 3.4 (1.8–6.3) and 2.8 (1.6–4.6), respectively, both *p* < 0.0001. The Kaplan–Meier curves for both endpoints are presented in Figure 1a,b. Among the biomarkers of interest: sTfR HR (95% CI): 1.4 (1.04–1.8), NGAL_(log)_ HR (95% CI): 2.0 (1.3–3.1) and GDF-15_(log)_ HR (95% CI): 4.0 (1.2–13.0) significantly impacted the one-year survival, all *p* < 0.05.

## 4. Discussion

The principal finding of our study is that de novo AHF and ADHF patients may present themselves with numerous comparable clinical characteristics, leading to the categorization of AHF as a single entity. However, several variances in biomarker profiles can be recognized in AHF, which may be linked to differentiation in the pathophysiology of the syndrome at different stages of the disease and prognosis [22]. Surprisingly, a few of the examined biomarkers did not reveal significant differences, which may actually be related to the fact that a number of changes may be more quantitative than qualitative during the course of the disease.

There were numerous similarities in the clinical and laboratory characteristics of de novo AHF and ADHF patients. In our study, age and dyspnea were equable between both groups, as were the parameters of hemoglobin, white blood cell and platelet counts. Interestingly, a previous meta-analysis has indicated that a lower age of de novo AHF patients is likely; however, we did not observe such an association [23]. The inflammatory biomarkers, like C-reactive protein, IL-6 and IL-22, were also comparable between both groups, alongside several remodeling markers. However, the ADHF patients were more likely to have a reduced LVEF and a greater likelihood of ischemic heart disease, whereas higher blood pressure was more prevalent in de novo AHF patients.

In terms of comorbidity, CAD was significantly more frequently observed in the ADHF group, whereas de novo patients were more likely to be hypertensive. ADHF patients were more likely to have been administered either an ACEI/ARB, beta-blocker or MRA prior to administration, and this trend continued during hospitalization.

We also showed that the organ functions differed between the two patient profiles, with ADHF likely to have worse organ functions in general. This observation was not surprising, as multiorgan dysfunction is a well-recognized marker of poor outcome in AHF [7,24,25,26,27,28]. It has been reported previously that elevated bilirubin is an ominous sign in heart failure [26,29,30,31,32]. In our study, we observed significantly higher values of total bilirubin at admission in ADHF patients. On the other hand, in terms of kidney function, the ADHF profile tended only to have higher levels of creatinine and higher blood urea nitrogen, yet serum sodium was at more favorable levels in de novo AHF.

Our recent paper showed that NTproBNP is a correlate of lactate, an organic acid produced during anerobic cell metabolism and a marker of perfusion mirroring the energetic challenges AHF presents to the body [33]. We also showed that elevated lactate at the admission of AHF patients without over hypoperfusion is a marker of a poor outcome [34]. The lactate levels of the ADHF group at all time points (admission, day 1 and discharge) were numerically higher than that of the de novo AHF patients, albeit only at discharge was the difference statistically significant. In order to better understand whether differences in the biomarkers between our two groups had an influence upon the subsequent course of the disease, we subsequently divided the patient cohort into two groups: those who went event-free through follow-up and those who experienced either death or HF rehospitalization. Lactate and NTproBNP were clearly associated with a poor outcome in that comparison.

A link between lactate and iron levels in HF has also recently been suggested, which is perhaps expected given the well-described role iron plays in cell energy production [35]. Soluble transferrin receptor (sTfR) concentrations are relative to the cellular iron demand, and raised levels are indicative of iron deficiency [36,37,38]. In our study, the ADHF group had significantly higher sTfR when compared to the de novo group, which may link iron metabolism with the natural progression of the disease.

Of the several remodeling markers we examined, we were surprised to see that only values for Intercellular Adhesion Molecule 1 (ICAM-1) presented a significant difference, as these values were increased in the ADHF group. ICAM-1 belongs to the immunoglobulin superfamily and enables the binding of leukocytes to endothelial cells [39]. Studies concerning cardiac remodeling have identified ICAM-1 as a critical component of leukocyte tissue infiltration in the left ventricle [14,39]; thus, our ICAM-1 results were expected, considering the ADHF group had a significantly impaired LVEF. Furthermore, we observed the opposite trend of higher follistatin levels in the de novo AHF patients. Follistatin is known to play a role in the inflammatory cascade, and as far as we are aware, no such studies exist concerning the relationship of follistatin and ICAM-1 in AHF. We were surprised to see that the remodeling markers between both AHF groups were generally alike. Future research is needed to evaluate the potential relationship between follistatin and ICAM-1; additionally, a wider evaluation of the remodeling biomarkers, such as copeptin, Monocyte Chemotactic Protein-3 and ST-2, and their roles in the patient profiles of de novo AHF and ADHF are needed.

Interestingly, when we evaluated the patient data in terms of those who went event-free or experienced our composite endpoint (death or rehospitalization), only lipocalin/NGAL was significantly different, with increased serum concentrations in the death or rehospitalization group. The ADHF group had significantly worse outcomes over one year when compared to the de novo group. This may be the result of compromised organ function and a worse baseline status (particularly, liver, kidney and left ventricular function) in the first group. Importantly, several of the biomarkers examined did influence the outcome in our population, such as: lactate, sTfR, Lipocalin/NGAL and GDF-15. Galectin-3 impacted upon the in-hospital mortality in our population. This marker has already been shown to impact the prognosis of ambulatory chronic HF patients [40]. On the other hand, we were surprised to see that some molecules (with a high theoretical/pathophysiological potential to be linked with disease progression and outcome) did not reveal any influence upon the patient outcomes [41,42,43,44].

This study was not without its limitations. Firstly, it was a single-center post hoc analysis with a limited sample size drawn only from the Polish resident population. Secondly, the analyzed biomarkers represent an area of possible selection bias, and finally, as with many similar studies, male patients were overrepresented. Moreover, the study was calculated and powered to reveal differences in many (i.e., bilirubin, lactate, NTproBNP, sTfR, ferritin, ICAM-1, IL-22, follistatin and MMP-9) but not all markers of interest; thus, one needs to take into consideration that the lack of the differences in some cases may actually be a result of study underpower. Confirmation of the differentiation between de novo AHF and ADHF is needed via multi-center studies and a larger number of patients.

## 5. Conclusions

Numerous similarities existed between the profiles of de novo AHF and ADHF. Age and dyspnea between both groups were comparable, alongside the inflammatory and nearly all remodeling biomarkers (with the exception of ICAM-1). However, only superficially is AHF a single entity, as during the natural course of AHF, the pathophysiology underlying patients may differ, and therefore, the distinct biomarker profiles of de novo AHF and ADHF can be discerned. In particular, organ function biomarkers (bilirubin, creatinine and blood urea) congestion and perfusion (NTproBNP and lactate) presented more unfavorably in ADHF alongside higher rates of CAD, resulting in a lower probability of survival. De novo AHF, an early stage of the disease, is more hypertensive in profile, with fewer complicating factors. Increased lipocalin/NGAL was associated with death or rehospitalization during follow-up.

## Figures and Tables

**Table 2:** Administered drug class and invasive procedures.

Drug Class or Procedure	Population	De Novo AHF	ADHF	*p*
Before hospitalization:				
ACEI/ARB	157 (63%)	48 (46%)	118 (82%)	<0.001
Beta-blocker	150 (61%)	43 (41%)	107 (74%)	<0.005
MRA	60 (24%)	10 (9.6%)	50 (35%)	<0.001
During hospitalization:				
Intravenous nitrates	129 (52%)	63 (61%)	66 (46%)	0.02
Inotropes	26 (10%)	6 (6%)	20 (14%)	0.03
ACEI/ARB	216 (87%)	91 (88%)	125 (87%)	0.87
Beta-blocker	233 (93%)	97 (93%)	136 (94%)	0.20
MRA	107 (43%)	45 (43%)	62 (43%)	0.97
Intravenous furosemide	248 (100%)	104 (100%)	144 (100%)	1.0
Invasive procedures:				
Thoracentesis	14 (6%)	4 (4%)	10 (7%)	0.27
Peritoneocentesis	6 (2%)	1 (1%)	5 (4%)	0.19
CPAP	19 (7%)	9 (9%)	9 (6%)	0.51

ACEI—Angiotensin-converting enzyme inhibitors, ARB—angiotensin receptor blockers, MRA—mineralocorticoid receptor antagonists and CPAP—continuous positive airway pressure.

**Table 4:** Comparison of the selected biomarkers between patients who experienced an event (death or heart failure rehospitalization, whichever occurred first) and the one-year event-free group.

Variable	Event-Free Patients	Death or Rehospitalization	*p*
Inflammation:			
C-reactive protein (mg/L)	6.4 (3.4–15.4)	11.0 (5.2–27.4)	0.014
IL-6 (pg/mL)	7.8 (0.5–16.0)	11.0 (0.5–30.3)	0.110
IL-22 (pg/mL)	5.5 (0.0–19.0)	8.0 (1.0–22.0)	0.345
WBC (G/L)	8.9 ± 3.4	9.8 ± 5.7	0.107
Liver function tests:			
AST at admission (IU/L)	30 (22–43)	27 (21–40)	0.447
ALT at admission (IU/L)	32 (22–57)	29 (21–50)	0.560
Total bilirubin at admission (mg/dL)	1.3 ± 1.1	1.5 ± 1.6	0.280
Perfusion and congestion markers:			
Lactate at admission (mmol/L)	2.1 ± 0.9	2.4 ± 1.5	0.042
Lactate at day-1 (mmol/L)	2.0 ± 0.6	2.3 ± 1.7	0.021
Lactate at discharge(mmol/L)	2.0 ± 0.8	1.9 ± 0.8	0.571
NTproBNP at admission (pg/mL)	5080 (2944–9101)	6312 (4083–13,944)	0.004
NTproBNP at discharge (pg/mL)	2636 (1499–4802)	4318 (2616–8210)	<0.0005
Remodeling and other markers:			
MMP-9 (ng/mL)	329.4 ± 310.8	354.6 ± 306.2	0.611
Follistatin (pg/mL)	3484.5 ± 1699.3	2508.0 ± 1729.4	0.916
Selectin (ng/mL)	32.8 ± 16.2	33.9 ± 18.5	0.647
Lipocalin/NGAL (ng/mL)	77.9 ± 51.8	95.7 ± 55.6	0.039
PF4 (ng/mL)	6692.4 ± 3543.3	6751.1 ± 3068.9	0.893
Myostatin (pg/mL)	1870.3 ± 1117.7	1691.2 ± 1108.3	0.218
ICAM-1 (ng/mL)	387.6 ± 171.9	409.5 ± 192.8	0.353
GDF-15 (pg/mL)	4640.4 ± 1598.1	4697.6 ± 1566.0	0.875
Galectin-3 (ng/mL)	19.0 (12.6–31.6)	21.9 (12.7–35.2)	0.56
Iron status:			
Fe (µg/dL)	58.0 ± 31.7	53.0 ± 26.7	0.223
Total iron binding capacity (µg/dL)	350.5 ± 71.5	341.0 ± 65.6	0.319
sTfR at admission (mg/L)	1.9 ± 0.7	2.2 ± 0.9	0.006
Ferritin (µg/L)	184.7 ± 151.6	164.3 ± 145.0	0.325

## Data Availability

All relevant data is contained within the manuscript.

## Supplementary Materials

The following are available online at https://www.mdpi.com/article/10.3390/biom11111701/s1: Table S1: Selected values of biomarkers—EF ≤ 40% vs. EF > 40%. Text S1: Brief biomarker overview.
