# Supplementary material for: Differences in the Biomarker Profile of De Novo Acute Heart Failure versus Decompensation of Chronic Heart Failure
Source: Biomolecules. 2021 Nov 16;11(11):1701. doi: 10.3390/biom11111701 (PMC8615401; doi:10.3390/biom11111701)
Supplement: Supplementary file 1 [file biomolecules-11-01701-s001.zip › biomolecules-1442747-supplementary.pdf]

Supplementary information:

Table S1. Selected values of biomarkers—EF≤ 40% vs. EF>40%

	EF≤ 40%	EF>40%	
	Mean±SD/Median [IQR]	Mean±SD/Median [IQR]	p
Urea (mg/dl)	59±27	61±38	0.518
Creatinine (mg/dl)	1.34±0.45	1.40±0.63	0.449
Total Bilirubin (mg/dl)	1.1 [0.7-1.7]	0.9 [0.7-1.5]	0.092
Lactate (mmol/l)	2.0 [1.5-2.5]	2.0 [1.5-2.7]	0.961
NTproBNP (pg/ml)	6312 [3742-13944]	5058 [2772-7189]	0.002
IL6 (pg/ml)	9.0 [0.8-19.8]	8.3 [0.5-21.7]	0.967
IL22 (pg/ml)	7.0 [1.0-22.0]	7.0 [1.0-18.0]	0.633
MMP-9 (ng/ml)	368.5±317.2	295.2±289.8	0.145
Follistatin (pg/ml)	2555.4±1558.5	2383.5±1952.4	0.45
Selectin (ng/ml)	32.8±16.8	34.0±17.8	0.585
Lipocalin/NGAL (ng/ml)	84.7±55.0	87.0±52.8	0.799
PF4 (ng/ml)	6550.4±3389.4	7012.7±3293.3	0.301
Myostatin (pg/ml)	1900.4±1192.2	1616.8±940.8	0.056
ICAM-1 (ng/ml)	403.2±182.6	383.9±176.8	0.423
GDF-15 (pg/ml)	4784.2±1447.6	4498.9±1746.2	0.437
CD40Ligand (ng/ml)	1.02±0.87	0.90±0.41	0.436
Galectin (ng/ml)	21.6 [11.7-31.6]	19.5 [13.7-35.4]	0.48
Fe (µg/dl)	0.5±26.5	55.4±34.9	0.865
TIBC (µg/dl)	346.2±62.3	347.0±80.3	0.936
UIBC (µg/dl)	290.1±56.9	291.6±88.3	0.879
Transferrin saturation coefficient (%)	16.1±6.9	16.8±11.8	0.617
Soluble transferrin receptor (mg/l)	2.0±0.8	2.0±0.9	0.757
Ferritin (µg/dl)	163.5±136.2	200.2±168.8	0.088

1. Brief biomarker overview

A current literature review was undertaken and biomarkers were selected on the basis of their relation to pertinent HF pathophysiological pathways. The selected biomarkers are involved in inflammation (CRP, IL-6, IL-22, WBC), liver function (AST, ALT, bilirubin), perfusion and congestion (NTproBNP, lactate) , iron status (Fe, TIBC, sTfR, Ferritin)and cardiac remodeling (MMP-3, Follistatin, Selectin, Lipocalin, PF4, Myostatin, ICAM -1, GDF-15, Galectin -3). The properties of each

marker and evidence in HF have been described elsewhere [33,38-44].So here we just shortly described each other.

Inflammation biomarkers are elevated in some inflammatory states. C-reactive protein is an acute phase protein produced in the liver in response to IL-6. CRP is increased in HF and higher levels are associated with features of more severe HF and are independently associated with mortality and morbidity, yet there remains limited data on its prognostic value in HF [1, 2]. IL-6 is one of the most important and multidirectional cytokines, which is secreted mainly by monocytes and macrophages . Il-6 can be useful in the prediction of HF onset or another cardiovascular events [3] IL-22 is a pro-inflammatory cytokine, which is involved in cardiovascular diseases . It is produced by T lymphocytes (T-helper lymphocytes, NKT lymphocytes, NK cells and non-specific lymphoid cells) . IL-22 improved Angiotensin II-induced cardiomyocyte apoptosis via inhibiting the intrinsic apoptosis pathway [4].

Both perfusion and congestion markers determine poor outcome . N-terminal proBNP (NT-proBNP) is a well established diagnostic and prognostic biomarker of congestion in HF. It is released by cardiac myocytes in response to stretch. The pro B-type natriuretic peptide is cleaved into the active C-terminal BNP (B-type natriuretic peptide) and an the inert NT-proBNP, both of which can be utilized as biomarkers. Several studies suggest that NT-proBNP levels can be used as an effective risk prediction tool in HF, regardless of ejection fraction status.[5,6]. Lactate is produced by aneorobic metabolism by most tissues, it is a metabolite of easy and quick assessment . Lactate may reflect inadequate tissue perfusion in conditions such as AHF. One purported mechanism for the development of lactic acidosis in heart failure is that when the cardiac output is no longer sufficient to supply adequate oxygen to the metabolizing tissues, and the ability of the peripheral tissues to increase the extraction of oxygen from the blood is exhausted, that tissue hypoxia ensues[7].

The matrix metalloproteinase (MMP) family is comprised of zinc-dependent endopeptidase enzymes , one of them is the gelatinase —MMP- 9 which is elevated under certain pathological conditions including multiple cardiovascular diseases.

MMP- 9 plays a role in atherosclerosis, hypertension, myocardial infarction (MI), and heart failure. In terms of cardiac remodeling, a recent study suggests MMP-9 may play both a positive and negative role, suggesting a more complete insight into MMP-9 substrates is required in order to better understand its role in HF and cardiac remodeling (8). MMP-9 is secreted by various cell types, including neutrophils, macrophages, and fibroblasts [9].

The glycoprotein Follistatin 1 (FSTL1) is secreted in various tissues including heart and skeletal. It has been shown to participate in regulating developmental

processes and to be involved in states of disease and injury. FSTL1 has been associated with left ventricular hypertrophy and recent findings concerning FSTL1 in acute coronary syndrome and heart failure suggest it may be a possible target for regenerative cardiac treatments [10,11].

Selectins are a family of cell adhesion molecule glycoproteins linked to an adverse role in the inflammation response and subsequent cardiac remodeling post myocardial infarction. Selectins are a key research area regarding anti-adhesive therapies aiming to reduce inflammatory injury [12].

Myostatin is known to be expressed in heart tissue and it's exact role in heart physiology and pathophysiology remains debated. A recent study has suggested that in the left-ventricle, myostatin signaling counters IGF-1 signaling in healthy hearts yet alterations to this countering are observable in end-stage heart failure - namely heightened myostatin protein activation [13].

ICAM1 is immunoglobulin-like adhesion molecule that mediates leukocyte arrest and transendothelial migration from the blood stream to the inflammation site. Studies have confirmed the major role ICAM-1 plays in cardiac remodeling in pressure-overload HF. Studies in mice models have found that ICAM-1 levels increased within 48 hours after myocardial infarction. ICAM1$^{-/-}$ mice are protected from HF in pressure-overload contexts [14].

Growth/Differentiation Factor-15 (GDF-15) is a proven biomarker in a variety of pathologies - including cardiovascular disease [15, 16]. Research has begun to indicate that GDF-15 plays a part in fibrosis, hypertrophy, and endothelial dysfunction. However, due to the complex nature of the GDF-15 protein, understanding the exact role GDF-15 has not yet been adequately described[17]. GDF-15 is known to be expressed by cardiomyocytes, endothelium, vascular smooth cells, macrophages and adipocytes [18].

Galectin-3, like all galectins, is a carbohydrate binding protein. Galectin-3 is expressed by a wide variety of cells [19] and increased expression has been associated with an increased likelihood of developing decompensated heart failure. Pathways have been suggested which propose Galectin-3 may actually be a 'culprit biomarker' and therefore a target for intervention[20]. Galectin-3 has been associated with inflammation, remodeling and renal function domains [15].

Neutrophil gelatinase-associated lipocalin (NGAL) is mainly known as a marker of renal function[21]. Lipocalin/NGAL plays a role in innate immunity and is expressed mainly by immune cells, hepatocytes, and renal tubular cells. It exerts bacteriostatic effects by interfering with iron processes of certain bacteria via iron sequestration [15,22]. Recent studies however, have challenged the status of

Lipocalin/NGAL as only a renal marker and have suggested its prognostic value in HF and as a potential therapeutic target. Increased serum Lipocalin/NGAL has been associated with LV dysfunction post myocardial infarction [23].

Iron status disruption is frequent amongst HF patients and has ominous prognostic consequences. Iron plays a key role in cell energy production [24] as a cofactor and enzyme catalyst. It is also crucial for erythropoiesis, oxygen transport, and tissue oxygenation [25], thus disrupted iron status may be representative of an increased energetic burden placed upon cardiac tissues during the course of heart failure, as we have previously suggested [26]. Iron status may be assessed via serum soluble transferrin receptor (sTfR) concentrations as sTfR concentrations are relative to cellular iron demand and raised levels are indicative of an increased demand for iron in the body [27] [28] [29]. Furthermore, Serum ferritin have been reported to be elevated in early-stage systolic HF [30].

Platelet factor 4, otherwise known as chemokine CXCL4, is released from the alpha-granules of activated platelets and promotes coagulation via inhibition of local antithrombin activity. Platelet factor 4 is suspected to play a role in cardiac remodeling, possibly through C-X-C Motif Chemokine Receptor 3. There is limited information currently available regarding platelet factor 4 and cardiac remodeling yet it remains an exciting area of potential future research [31].

References

[1] Geenen LW, Baggen VJM, van den Bosch AE, et al. Prognostic value of C-reactive protein in adults with congenital heart disease. Heart 2021;107:474-481.

[2] Tang WH, Shrestha K, Van Lente F, et al. Usefulness of C-reactive protein and left ventricular diastolic performance for prognosis in patients with left ventricular systolic heart failure. Am J Cardiol. 2008;101:370-3.

[3] Nishida H, Horio T, Suzuki Y, Iwashima Y, Tokudome T, Yoshihara F, Nakamura S, Kawano Y. Interleukin-6 as an independent predictor of future cardiovascular events in high-risk Japanese patients: comparison with C-reactive protein. Cytokine. 2011 Mar;53(3):342-6. doi: 10.1016/j.cyto.2010.12.005.

[4] Interleukin-22 alleviates Angiotensin II-induced cardiomyocyte apoptosis by preventing the intrinsic mitochondrial pathway Lin Xia, Yang Che and Zhaoliang Su J Immunol May 1, 2020, 204 (1 Supplement) 59.37;

[5] B-type natriuretic peptide and prognosis in heart failure patients with preserved and reduced ejection fraction. van Veldhuisen DJ, Linssen GC, Jaarsma T, van Gilst WH, Hoes AW, Tijssen JG, Paulus WJ, Voors AA, Hillege HL. 14, 2013, Journal of the American College of Cardiology, Vol. 61, pp. 1498-1506.

[6] Prognosis and NT-proBNP in heart failure patients with preserved versus reduced ejection fraction. Salah K, Stienen S, Pinto YM, et al. 2019, Heart, Vol. 105, pp. 1182-1189

[7] Adamo L, Nassif ME, Novak E, LaRue SJ, Mann DL. Prevalence of lactic acidaemia in patients with advanced heart failure and depressed cardiac output. Eur J Heart Fail. 2017 Aug;19(8):1027-1033. doi: 10.1002/ejhf.628. Epub 2016 Sep 20.

[8] Iyer RP, Jung M, Lindsey ML. MMP-9 signaling in the left ventricle following myocardial infarction. Am J Physiol Heart Circ Physiol. 2016;311(1):H190-H198. doi:10.1152/ajpheart.00243.2016

[9] Yabluchanskiy A, Ma Y, Iyer RP, Hall ME, Lindsey ML. Matrix metalloproteinase-9: Many shades of function in cardiovascular disease. Physiology (Bethesda). 2013;28(6):391-403. doi:10.1152/physiol.00029.2013

[10] Tanaka K, Valero-Muñoz M, Wilson RM, et al. Follistatin like 1 Regulates Hypertrophy in Heart Failure with Preserved Ejection Fraction. JACC Basic Transl Sci. 2016;1(4):207-221. doi:10.1016/j.jacbts.2016.04.00

[11] Peters MMC, Meijs TA, Gathier W, Doevendans PAM, Sluijter JPG, Chamuleau SAJ, Neef K. Follistatin-like 1 in Cardiovascular Disease and Inflammation. Mini Rev Med Chem. 2019;19(16):1379-1389. doi: 10.2174/1389557519666190312161551.

[12] Weil BR, Neelamegham S. Selectins and Immune Cells in Acute Myocardial Infarction and Post-infarction Ventricular Remodeling: Pathophysiology and Novel Treatments. Front Immunol. 2019;10:300. Published 2019 Feb 27. doi:10.3389/fimmu.2019.00300

[13] Baán, J.A., Varga, Z.V., Leszek, P. et al. Myostatin and IGF-I signaling in end-stage human heart failure: a qRT-PCR study. J Transl Med 13, 1 (2015). https://doi.org/10.1186/s12967-014-0365-0

[14] Salvador AM, Nevers T, Velázquez F, Aronovitz M, Wang B, Abadía Molina A, Jaffe IZ, Karas RH, Blanton RM, Alcaide P. Intercellular Adhesion Molecule 1 Regulates Left Ventricular Leukocyte Infiltration, Cardiac Remodeling, and Function in Pressure Overload-Induced Heart Failure. J Am Heart Assoc. 2016 Mar 15;5(3):e003126. doi: 10.1161/JAHA.115.003126.

[15] Demissei BG, Valente MA, Cleland JG, O'Connor CM, Metra M, Ponikowski P, Teerlink JR, Cotter G, Davison B, Givertz MM, Bloomfield DM, Dittrich H, van der Meer P, van Veldhuisen DJ, Hillege HL, Voors AA. Optimizing clinical use of biomarkers in high-risk acute heart failure patients. Eur J Heart Fail. 2016 Mar;18(3):269-80. doi: 10.1002/ejhf.443. Epub 2015 Dec 3. PMID: 26634889.

[16] Growth differentiation factor 15 predicts poor prognosis in patients with heart failure and reduced ejection fraction and anemia: results from RED-HF Thor Ueland, Lars Gullestad, Lei Kou, James B. Young, Marc A. Pfeffer, Dirk Jan van Veldhuisen, Karl Swedberg, John J. V. Mcmurray, Akshay S. Desai, Inderjit S. Anand & Pål Aukrust

[17] Wesseling, M., de Poel, J. H. C., and de Jager, S. C. A. (2020) Growth differentiation factor 15 in adverse cardiac remodelling: from biomarker to causal player. ESC Heart Failure, 7: 1488–1501. https://doi.org/10.1002/ehf2.12728.

[18] Kempf T, Zarbock A, Widera C, et al. GDF-15 is an inhibitor of leukocyte integrin activation required for survival after myocardial infarction in mice. Nat Med. 2011;17:581-8.

[19] Henderson NC, Mackinnon AC, Farnworth SL, et al. Galectin-3 expression and secretion links macrophages to the promotion of renal fibrosis. Am J Pathol. 2008;172:288-98.

[20] de Boer RA, Yu L, van Veldhuisen DJ. Galectin-3 in cardiac remodeling and heart failure [published correction appears in Curr Heart Fail Rep. 2012 Sep;9(3):163]. Curr Heart Fail Rep. 2010;7(1):1-8. doi:10.1007/s11897-010-0004-x

[21] Haase M, Devarajan P, Haase-Fielitz A, et al. The outcome of neutrophil gelatinase-associated lipocalin-positive subclinical acute kidney injury: A multicenter pooled analysis of prospective studies. J Am Coll Cardiol. 2011;57:1752-61.

[22] Schmidt-Ott KM, Mori K, Li JY, et al. Dual action of neutrophil gelatinase-associated lipocalin. J Am Soc Nephrol. 2007;18:407-13.

[23] Martínez-Martínez E, Buonafine M, Boukhalfa I, Ibarrola J, Fernández-Celis A, Kolkhof P, Rossignol P, Girerd N, Mulder P, López-Andrés N, Ouvrard-Pascaud A, Jaisser F. Aldosterone Target NGAL (Neutrophil Gelatinase-Associated Lipocalin) Is Involved in Cardiac Remodeling After Myocardial Infarction Through NFκB Pathway. Hypertension. 2017 Dec;70(6):1148-1156. doi: 10.1161/HYPERTENSIONAHA.117.09791.

[24] Iron Metabolism Contributes to Prognosis in Coronary Artery Disease: Prognostic Value of the Soluble Transferrin Receptor Within the AtheroGene Study. Henri Weidmann PhD, Johannes H. Bannasch MD , et al. 9, 2020, Journal of the American Heart Association, Vol. 9.

[25]Jankowska EA, von Haehling S, Anker SD, et al. Iron deficiency and heart failure: diagnostic dilemmas and therapeutic perspectives. Eur Heart J. 2013; 34(11): 816–829, doi: 10.1093/eurheartj/ehs224, indexed in Pubmed: 23100285
[26]Biegus J, Zymliński R, Sokolski M, Jankowska EA, Banasiak W, Ponikowski P. Elevated lactate in acute heart failure patients with intracellular iron deficiency as identifier of poor outcome. Kardiol Pol. 2019;77(3):347-354. doi: 10.5603/KP.a2019.0014. Epub 2019 Feb 11.
[27]Effects of exercise on soluble transferrin receptor and other variables of the iron status. Schumacher YO, Schmid A, König D, et al. 36, 2002, British Journal of Sports Medicine, pp. 195-199.
[28]Adjusting soluble transferrin receptor concentrations for inflammation: Biomarkers Reflecting Inflammation and Nutritional Determinants of Anemia (BRINDA) project. Rohner F, Namaste SM, Larson LM, et al. 1, 2017, Am J Clin Nutr, Vol. 106, pp. 372S-382S.
[29]Serum transferrin receptor: a quantitative measure of tissue iron deficiency. Skikne BS, Flowers CH, Cook JD. 75, May 1990, Blood, Vol. 1, pp. 1870-6.
[30]Iron status in patients with chronic heart failure . Ewa A Jankowska 1, Jolanta Malyszko, Hossein Ardehali, Ewa Koc-Zorawska, Waldemar Banasiak, Stephan von Haehling, Iain C Macdougall, Guenter Weiss, John J V McMurray, Stefan D Anker, Mihai Gheorghiade, Piotr Ponikowski
[31]Chemokines in cardiac fibrosis. Ruoshui Li, Nikolaos G Frangogiannis. Current Opinion in Physiology.Volume 19. 2021. doi: /10.1016/j.cophys.2020.10.004
